# Supplementary material for: Pyruvate Dehydrogenase Kinase 1 inhibition mediated oxidative phosphorylation enhancement in cartilage promotes osteoarthritis progression
Source: BMC Musculoskelet Disord. 2023 Jul 20;24:597. doi: 10.1186/s12891-023-06585-6 (PMC10357736; doi:10.1186/s12891-023-06585-6)
Supplement: Supplementary file 1 — Additional file 1. [file 12891_2023_6585_MOESM1_ESM.zip › BP.Gsea.1653623667859/GOBP_NEGATIVE_REGULATION_OF_B_CELL_PROLIFERATION.html]

Details for gene set GOBP\_NEGATIVE\_REGULATION\_OF\_B\_CELL\_PROLIFERATION[GSEA]

|  || Dataset | 55457\_collapsed\_to\_symbols.cls.cls #OA\_versus\_normal.cls.cls #OA\_versus\_normal\_repos |
| Phenotype | cls.cls#OA\_versus\_normal\_repos |
| Upregulated in class | OA |
| GeneSet | GOBP\_NEGATIVE\_REGULATION\_OF\_B\_CELL\_PROLIFERATION |
| Enrichment Score (ES) | 0.49598214 |
| Normalized Enrichment Score (NES) | 1.4871923 |
| Nominal p-value | 0.06912442 |
| FDR q-value | 0.39182273 |
| FWER p-Value | 1.0 |
Table: GSEA Results Summary

  

Fig 1: Enrichment plot: GOBP\_NEGATIVE\_REGULATION\_OF\_B\_CELL\_PROLIFERATION      
 Profile of the Running ES Score & Positions of GeneSet Members on the Rank Ordered List

  

| SYMBOL | TITLE | RANK IN GENE LIST | RANK METRIC SCORE | RUNNING ES | CORE ENRICHMENT || 1 | CD300A | CD300a molecule [Source:HGNC Symbol;Acc:HGNC:19319] | 730 | 0.587 | 0.0783 | Yes |
| 2 | BTK | Bruton tyrosine kinase [Source:HGNC Symbol;Acc:HGNC:1133] | 889 | 0.545 | 0.1916 | Yes |
| 3 | FCGR2B | Fc fragment of IgG receptor IIb [Source:HGNC Symbol;Acc:HGNC:3618] | 2016 | 0.360 | 0.1870 | Yes |
| 4 | TYROBP | transmembrane immune signaling adaptor TYROBP [Source:HGNC Symbol;Acc:HGNC:12449] | 2058 | 0.355 | 0.2656 | Yes |
| 5 | TNFRSF21 | TNF receptor superfamily member 21 [Source:HGNC Symbol;Acc:HGNC:13469] | 2228 | 0.336 | 0.3297 | Yes |
| 6 | PKN1 | protein kinase N1 [Source:HGNC Symbol;Acc:HGNC:9405] | 2540 | 0.298 | 0.3743 | Yes |
| 7 | BLK | "BLK proto-oncogene, Src family tyrosine kinase [Source:HGNC Symbol;Acc:HGNC:1057]" | 2692 | 0.280 | 0.4270 | Yes |
| 8 | INPP5D | inositol polyphosphate-5-phosphatase D [Source:HGNC Symbol;Acc:HGNC:6079] | 3085 | 0.241 | 0.4520 | Yes |
| 9 | CASP3 | caspase 3 [Source:HGNC Symbol;Acc:HGNC:1504] | 3202 | 0.230 | 0.4960 | Yes |
| 10 | MNDA | myeloid cell nuclear differentiation antigen [Source:HGNC Symbol;Acc:HGNC:7183] | 6100 | -0.013 | 0.2741 | No |
| 11 | ATM | ATM serine/threonine kinase [Source:HGNC Symbol;Acc:HGNC:795] | 6179 | -0.019 | 0.2724 | No |
| 12 | LYN | "LYN proto-oncogene, Src family tyrosine kinase [Source:HGNC Symbol;Acc:HGNC:6735]" | 6514 | -0.043 | 0.2564 | No |
| 13 | TNFRSF13B | TNF receptor superfamily member 13B [Source:HGNC Symbol;Acc:HGNC:18153] | 8664 | -0.206 | 0.1369 | No |
| 14 | PAWR | pro-apoptotic WT1 regulator [Source:HGNC Symbol;Acc:HGNC:8614] | 8766 | -0.213 | 0.1782 | No |
| 15 | IL10 | interleukin 10 [Source:HGNC Symbol;Acc:HGNC:5962] | 8855 | -0.221 | 0.2223 | No |
| 16 | CTLA4 | cytotoxic T-lymphocyte associated protein 4 [Source:HGNC Symbol;Acc:HGNC:2505] | 10739 | -0.398 | 0.1677 | No |
Table: GSEA details [plain text format]

  

Fig 2: GOBP\_NEGATIVE\_REGULATION\_OF\_B\_CELL\_PROLIFERATION      
 Blue-Pink O' Gram in the Space of the Analyzed GeneSet

  

Fig 3: GOBP\_NEGATIVE\_REGULATION\_OF\_B\_CELL\_PROLIFERATION: Random ES distribution      
 Gene set null distribution of ES for **GOBP\_NEGATIVE\_REGULATION\_OF\_B\_CELL\_PROLIFERATION**
